# The relative role of patient physiology and device optimisation in cardiac resynchronisation therapy: A computational modelling study

**DOI:** 10.1016/j.yjmcc.2015.10.026

**Published:** 2016-07

**Authors:** Andrew Crozier, Bojan Blazevic, Pablo Lamata, Gernot Plank, Matthew Ginks, Simon Duckett, Manav Sohal, Anoop Shetty, Christopher A. Rinaldi, Reza Razavi, Nicolas P. Smith, Steven A. Niederer

**Affiliations:** a Division of Imaging Sciences and Biomedical Engineering, King's College London, United Kingdom; b Institute of Biophysics, Medical University of Graz, Austria; c Department of Cardiology, Guy's and St. Thomas' Hospital, London, United Kingdom; d Faculty of Engineering, University of Auckland, New Zealand

**Keywords:** AHR, acute haemodynamic response, CRT, cardiac resynchronisation therapy, DCM, dilated cardiomyopathy, ECG, electrocardiogram, EF, ejection fraction, EP, electrophysiology, HF, heart failure, LBBB, left bundle branch block, LV, left ventricle, MRI, magnetic resonance imaging, NCM, non-contact mapping, QRSd, QRS duration, RV, right ventricle, SR, sinus rhythm, TAT, total activation time of the ventricles, VPC, virtual patient cohort, Heart failure, Cardiac resynchronisation therapy, Dyssynchronous heart failure, Computational modelling, Patient-specific modelling

## Abstract

Cardiac resynchronisation therapy (CRT) is an established treatment for heart failure, however the effective selection of patients and optimisation of therapy remain controversial. While extensive research is ongoing, it remains unclear whether improvements in patient selection or therapy planning offers a greater opportunity for the improvement of clinical outcomes. This computational study investigates the impact of both physiological conditions that guide patient selection and the optimisation of pacing lead placement on CRT outcomes. A multi-scale biophysical model of cardiac electromechanics was developed and personalised to patient data in three patients. These models were separated into components representing cardiac anatomy, pacing lead location, myocardial conductivity and stiffness, afterload, active contraction and conduction block for each individual, and recombined to generate a cohort of 648 virtual patients. The effect of these components on the change in total activation time of the ventricles (ΔTAT) and acute haemodynamic response (AHR) was analysed. The pacing site location was found to have the largest effect on ΔTAT and AHR. Secondary effects on ΔTAT and AHR were found for functional conduction block and cardiac anatomy. The simulation results highlight a need for a greater emphasis on therapy optimisation in order to achieve the best outcomes for patients.

## Highlights

• Ventricular conduction block indicates patient response to CRT.
• Placement of CRT pacing leads strongly affects response to therapy.
• Improved treatment planning should be prioritised in order to maximise CRT outcomes.

## 1 Introduction

Heart failure (HF) is a significant disease in the western world, affecting 1 − 2% of the adult population and accounting for around 2% of all healthcare spending [1]. Cardiac Resynchronisation Therapy (CRT) is an effective treatment for dyssynchronous HF [2], reducing the risk of hospitalisation and death, and leading to improved heart function and quality of life [2], [3], [4]. However, using the current selection criteria of left ventricle (LV) dysfunction (LV ejection fraction (EF) ≤ 35%) and electrical dyssynchrony (QRS duration (QRSd) 120 − 149 with left bundle branch block (LBBB) or QRSd ≥ 150) [5], only 67% of patients benefit from CRT, while 39% of patients receiving standard pharmacological therapy improve without CRT [6].

The improvement of patient selection for CRT is therefore an important area of research, so that optimal outcomes for patients are achieved without unnecessary application of this expensive and invasive therapy. Patient physiology and demographic factors such as sex [7], diabetes [8] and ischaemia [9] have been shown to affect the potential benefits of CRT. Clinical research has also demonstrated the potential for improved response to CRT by optimisation of pacing lead location [10], [11], [12].

In this paper, we investigate whether either of the confounding roles of patient physiology or optimisation of CRT pacing lead location has a greater influence on patient outcomes. To systematically quantify the impact of each mechanism on patient response, we adopt a patient-specific computational modelling approach. Personalised biophysical modelling of cardiac function has increasingly been used to investigate the mechanisms underlying CRT response, and demonstrate its potential for advanced treatment planning [13], [14], [15], [16], [17]. We have constructed personalised and validated computational models of three CRT patients. These models were used to generate a virtual patient cohort, in which the relative impact of patient physiology and device placement on the total activation time (TAT) of the ventricles and acute haemodynamic response (AHR) was evaluated *in silico*.

## 2 Methods

### 2.1 Model development and personalisation

Each model consists of a biventricular, weakly coupled [18] model of cardiac electromechanics, combining a monodomain model of tissue electrophysiology using the ten Tusscher model of cellular electrophysiology [19] with a model of large deformation mechanics using the Guccione passive material law [20], a phenomenological model of myocardial active tension [16] and a 3 element Windkessel model of afterload. The complete modelling framework is described in detail in Section 1 of the online supplement accompanying this article. Models were personalised using clinical data, as outlined below and described in detail in Section 2 of the supplement.

#### 2.1.1 Cardiac anatomy and fibres

A tricubic Hermite description of the cardiac anatomy was fitted to a manually or automatically segmented end diastolic magnetic resonance imaging (MRI), as described previously [21], [22]. A generic fibre field based on *a priori* knowledge was mapped to each patient-specific anatomical model [16], and an additional high resolution tetrahedral discretisation was generated from each cubic Hermite mesh for the purposes of simulation of electrophysiology. Section 2.1 of the online supplement describes this process in detail.

#### 2.1.2 Electrical activation

Sinus rhythm (SR) activation was simulated by applying a stimulus current at the earliest sites of activation in the septum and RV as determined from ECG and EnSite™ non-contact mapping (NCM) data, and from the literature [23] in the case of the location of the earliest activation in the RV. The timings of these intrinsic stimuli were determined relative to the time of sinoatrial depolarisation as a reference point. CRT activation was simulated by adding electrical stimuli at pacing lead locations in the RV apex and LV epicardium determined from angiograms registered with MRI [24]. Following the clinical protocol, leads were paced 100 ms after sinoatrial node activation, prior to the activation of the intrinsic stimuli. Conduction block was included by means of a region of low conductivity, with its location determined from NCM. Activation sequences were validated by comparison with NCM activation maps. For additional details on the above steps, see Section 2.2 of the online supplement.

#### 2.1.3 Contraction

Simulation of the full cardiac cycle was carried out at SR and with CRT by mapping simulated depolarisation times to the cubic Hermite computational mesh, where they are used as an input to the active tension model. 2 parameters from the passive material law, 6 parameters from the active tension model and the 3 Windkessel model parameters were fitted to patient-specific pressure-volume (PV) loops at sinus rhythm and to the observed acute haemodynamic response (AHR) in the case of the active tension model. Mechanical contraction was validated by comparison with short axis cine MRI at sinus rhythm. Section 2.3 of the online supplement describes this process in detail.

#### 2.1.4 Patient cases

The personalisation workflow summarised above and described in detail in the online supplement was applied to three patients with demographics and baseline characteristics as shown in Table 1. All patients had a dilated cardiomyopathy (DCM) aetiology and NYHA class of III.

#### 2.1.5 Software tools

Simulations of cardiac electrophysiology were run with the Cardiac Arrhythmia Research Package (CARP) (http://carp.medunigraz.at/), developed at the Medical University of Graz (Graz, Austria) and the University of Bordeaux (Bordeaux, France). Simulations were performed on ARCHER (http://www.archer.ac.uk/), the UK national high performance computing (HPC) resource, using 288 cores and requiring 2.5 − 4 h execution time per model. Large deformation mechanics were simulated using CMISS (http://www.cmiss.org/), developed at the University of Auckland (Auckland, New Zealand), and executed on the HPC resource at the Department of Biomedical Engineering at King's College London, using 4 cores and requiring 14 − 20 h execution time per model.

### 2.2 Virtual patient cohort

A virtual patient cohort (VPC) was generated from combinations of discrete model components from each of the three personalised models. In the first virtual patient cohort (VPC1), model inputs were separated into 6 components: model geometry, including both the cardiac anatomy and the location of the pacing leads; tissue conductivity; the presence of conduction block; tissue stiffness; Windkessel model; and active tension model parameters. Generating models for all possible combinations of these 6 components resulted in a VPC of 486 models (2 possible values for the binary ‘presence of conduction block’ component, and 3 possible values for the 5 other components).

As VPC1 did not allow the differentiation of the effects of cardiac anatomy and CRT pacing lead location, a supplementary second virtual cohort (VPC2) was generated. In VPC2, pacing lead locations and cardiac anatomy were considered independently, while the tissue stiffness, Windkessel model and active tension model parameters for each patient were combined into a single ‘mechanical model parameters’ component to retain computational tractability of the VPC as a whole. This resulted in 5 components: cardiac anatomy; tissue conductivity; conduction block; pacing lead location; and mechanical model parameters. The VPC generated from all potential combinations of these components contained 162 models (2 possible values for the ‘presence of conduction block’ component, and 3 possible values for the others as above).

The pacing lead locations were readily mapped between anatomies in VPC2 by use of the cubic Hermite mesh. These structured meshes had a consistent topology, and so pacing locations were mapped between meshes by first evaluating their position in the internal local finite element coordinate space of the mesh of the source case, then reevaluating the Cartesian coordinate in the mesh of the destination case.

#### 2.2.1 Simulations

Simulations of cardiac contraction were performed for all of the models in both VPCs at SR and with CRT. This required a total of 36 simulations of electrophysiology (EP) and 972 simulations of large deformation mechanics for VPC1, and 108 simulations of EP and 324 simulations of mechanics for VPC2. The number of EP simulations required was smaller than the number of mechanics simulations in both VPCs, as the tissue stiffness, Windkessel model and active tension model have no effect on EP in our weakly coupled framework. In total, 223 thousand core hours were utilised on ARCHER and the Department of Biomedical Engineering HPC while executing these simulations.

#### 2.2.2 Metric calculation

Two metrics were calculated for each model: the fractional change in the total activation time of the ventricles (TAT) on pacing (ΔTAT), and the acute haemodynamics response (AHR). Changes in TAT are considered clinically important, as electrical resynchronisation of the ventricles by CRT is expected to be measured by a decrease in TAT. AHR is the fractional increase in the maximum rate of pressure development in the LV on pacing, and has been used in the clinic as a measure of acute response to therapy and as a predictor of long term remodelling [25]. These metrics are both relevant to the functional changes expected to be brought on by CRT, and are directly comparable to clinical studies. Both metrics were calculated for each model generated in VPC1 and VPC2 above.

### 2.3 Detection of effects

Differences caused by individual model components were identified by dividing a VPC into groups by the source of a given component, and identifying important differences in the above clinical metrics between these groups. Since VPC1 and VPC2 were not samples of a normally distributed population, conventional statistical tests of such changes were not applicable. Instead, boxplots of ΔTAT and AHR were plotted for each group, and inter-group differences caused by the component in question identified by either

• two groups having a non-overlapping inter-quartile range (IQR); or
• > 30% of data points of one group lying outside the boxplot whiskers of another group.
  Boxplots were plotted with the whiskers extending to 1.5 times the IQR past the first and third quartiles. Beyond the whiskers, data points are considered outliers and are plotted individually. Correlations between ΔTAT and AHR were evaluated with the non-parametric Spearman's rank correlation coefficient.

## 3 Results

### 3.1 Model personalisation

**Fig. 1:**
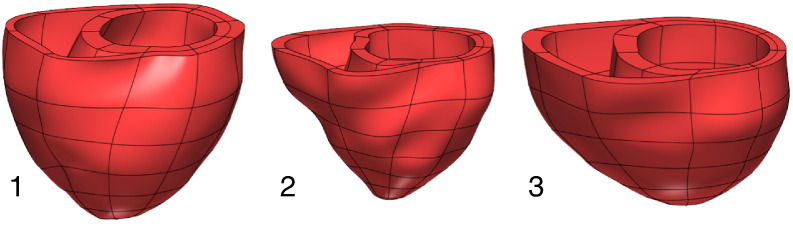
Personalised finite element meshes, derived from end Diastolic MRI, for the three patient cases in this study.

**Fig. 2:**
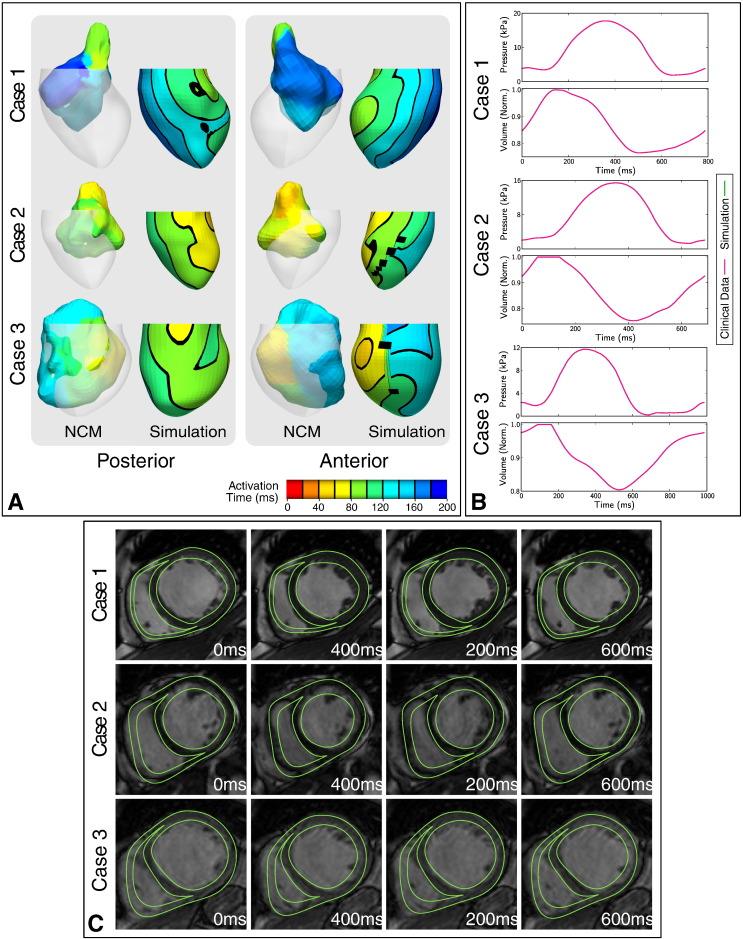
Parameter fitting and validation. (A) Tissue conductivity was fitted to sinus rhythm QRS duration and validated by visual comparison of simulated endocardial activation time maps (right) with EnSite non-contact mapping (NCM) data (left). Contours on the simulated activation time maps are displayed at 20 intervals, however these are not shown on the non-smooth maps from NCM, which are displayed registered with the MRI-based anatomical model for reference. (B) Tissue stiffness, Windkessel model and active tension model parameters were fitted to patient-specific pressure-volume data at sinus rhythm and (C) validated by comparison of simulated deformations with short axis cine MRI.

Anatomical models were generated from MRI for three patient cases, and are shown in Fig. 1. Fig. 2 summarises the parameter fitting and validation of 3 models of cardiac electromechanics to patient data using our personalisation workflow as described above.

The electrical conductivity parameters of each case were fitted to the QRS duration at SR, and validated by comparison of LV endocardial activation time maps with NCM (Fig. 2a). Due to discrepancies between the NCM-derived and MRI-derived endocardial geometries, and the application of the system in ventricles larger than its validated range [26], it was not possible to perform a quantitative validation of the simulated activation time maps. However, a qualitative comparison showed that the model provides a good representation of the endocardial activation pattern in all cases.

Passive tissue stiffness, Windkessel model parameters and active tension model parameters were fitted to patient-specific PV data at SR and AHR with CRT (Fig. 2b), and validated by comparison of predicted myocardial deformations with short axis cine MRI (Fig. 2c).

### 3.2 Virtual patient cohort

Virtual patient cohorts were generated as outlined in Section 2.2, and the fractional change in TAT due to pacing (ΔTAT) and the AHR were evaluated for each model.

#### 3.2.1 VPC1

**Fig. 3:**
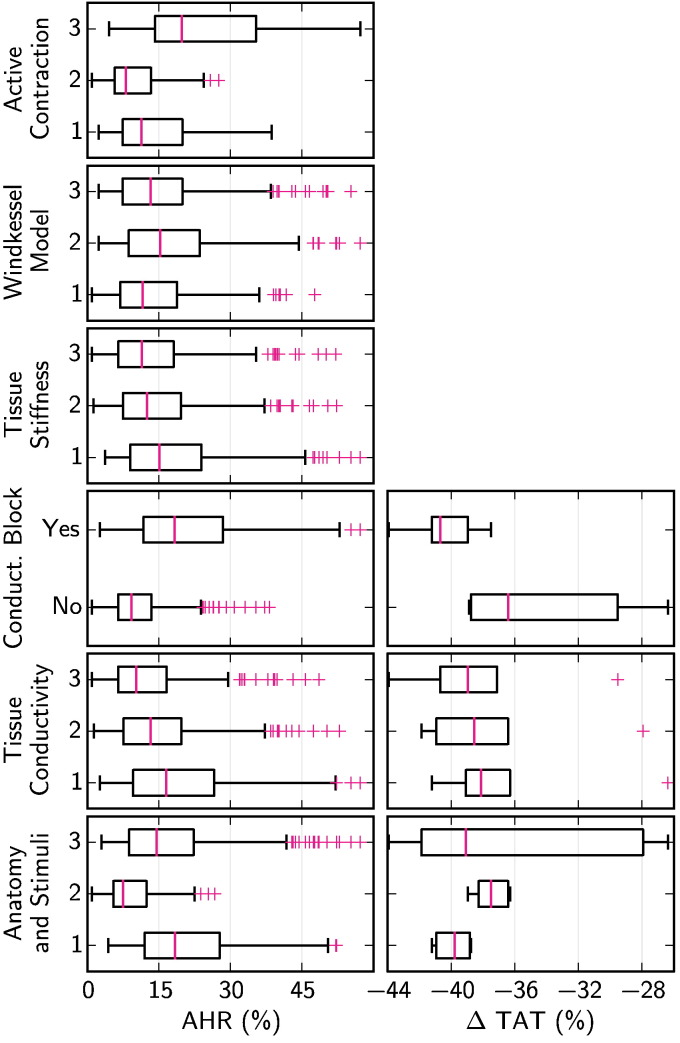
Boxplots showing the distribution of changes in TAT due to pacing and in AHR, when the first virtual patient cohort (VPC1) was separated into subgroups by each of 6 model components. Boxplots are not shown for changes in TAT for the tissue stiffness, Windkessel model and active contraction model components, as these components have no effect on the simulation of cardiac electrophysiology. Boxplot whiskers extend 1.5 times the IQR past the first and third quartiles, and outlying points are shown with magenta crosses.

Fig. 3 shows the distribution of ΔTAT and AHR for each model in the first virtual patient cohort (VPC1), separated into subgroups by each model component.

Based on the criteria outlined in Section 2.3, the cardiac anatomy and pacing lead location, tissue conductivity and conduction block were all found to have an important effect on changes in TAT due to pacing. Similarly, the cardiac anatomy and pacing lead location, conduction block and active contraction model parameters were found to have an important effect on AHR. The importance of tissue conductivity in ΔTAT was not repeated with AHR.

**Fig. 4:**
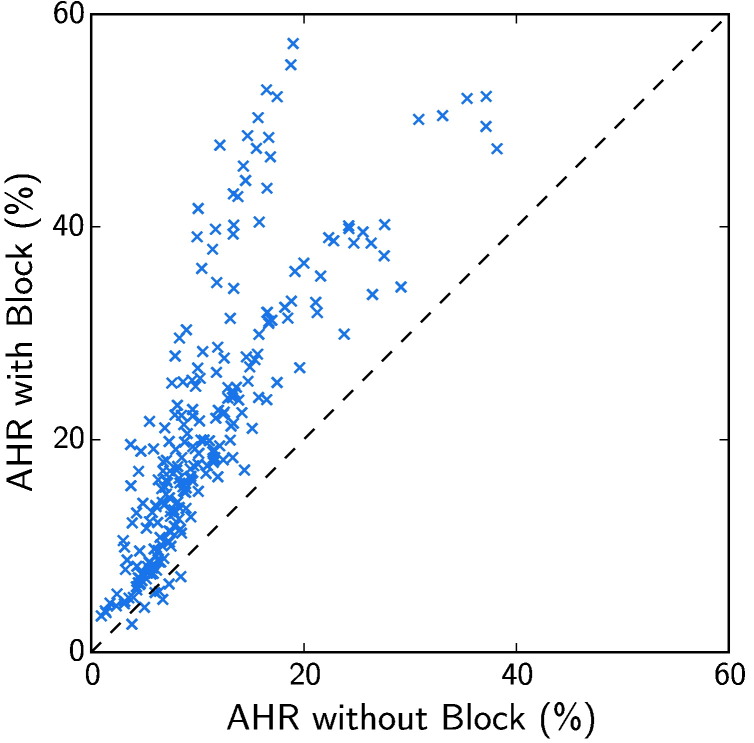
Comparison of AHR with and without conduction block for otherwise equivalent models in the first virtual patient cohort (VPC1). Most models can be seen to lie above the line of unity, with 97.1% having a better AHR when conduction block was present.

The differences in ΔTAT and AHR due to the presence of conduction block were particularly notable. Fig. 4 compares the AHR with and without conduction block for otherwise equivalent models in VPC1. This figure shows that, despite a large variation in AHR within the virtual cohort, 97.1% of otherwise equivalent models had a greater AHR with conduction block than without.

The results also showed that the cardiac anatomy and pacing lead location had an important effect on both changes in TAT with pacing and AHR. The separate contribution of each of these two factors is studied in VPC2, as reported in the next section.

#### 3.2.2 VPC2

**Fig. 5:**
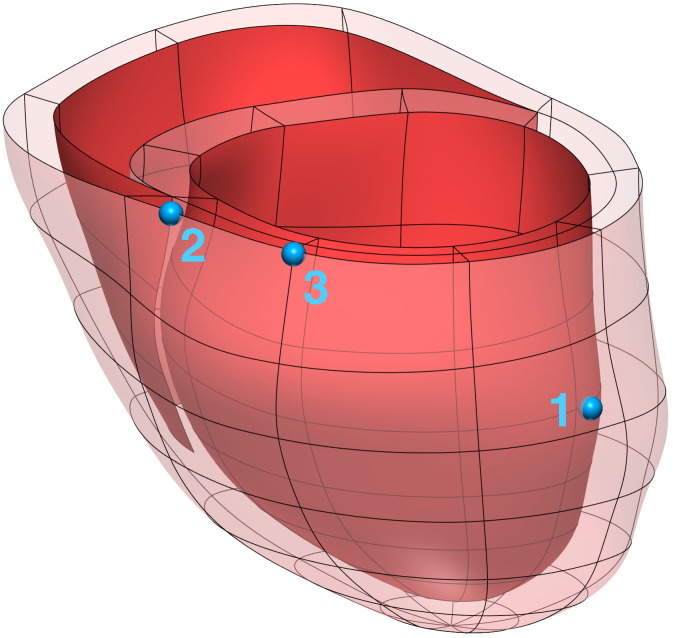
The location of the LV pacing lead was mapped to an equivalent anatomic position in other cases by use of the fitted cubic Hermite mesh, as described in Section 2.2. Here the pacing lead locations (blue, numbers correspond to lead source case) are shown mapped to the anatomy of case 3.

LV pacing lead locations were mapped between patient anatomies as described in Section 2.2, and are shown mapped to a single case in Fig. 5.

**Fig. 6:**
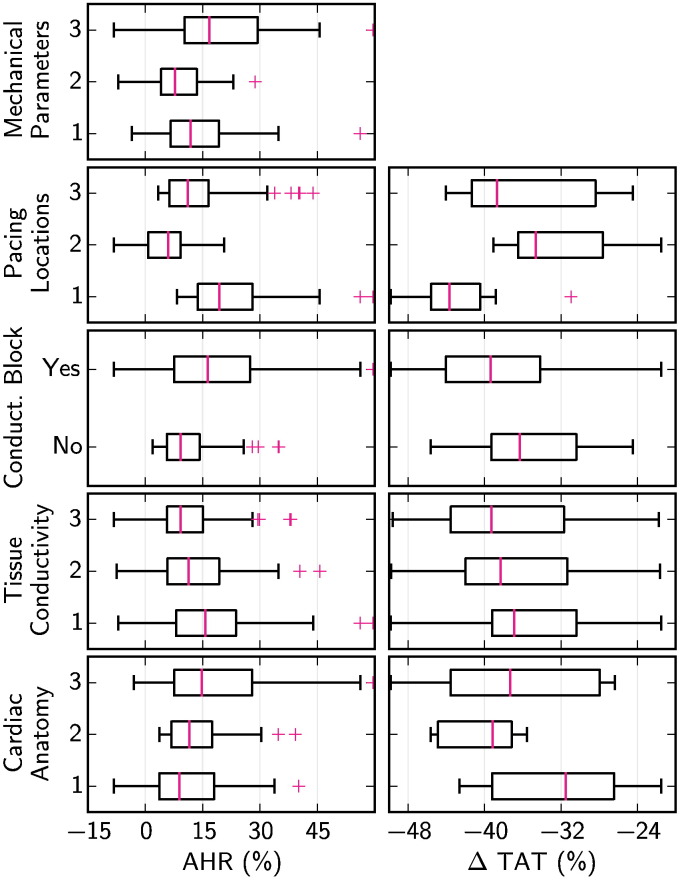
Boxplots showing the distribution of changes in TAT due to pacing and in AHR, when the second virtual patient cohort (VPC2) was separated into subgroups by each of 5 model components. Boxplots are not shown for changes in TAT for the mechanical model component, as it has no effect on the simulation of cardiac electrophysiology. Boxplot whiskers extend 1.5 times the IQR past the first and third quartiles, and outlying points are shown with magenta crosses.

Fig. 6 shows the distribution of ΔTAT and AHR for each model in the second virtual patient cohort (VPC2), separated into subgroups by model component.

Based on the criteria outlined in Section 2.3, both the cardiac anatomy and pacing lead location were found to have an important effect on changes in TAT due to pacing. Cardiac anatomy, conduction block, pacing lead location and mechanical model parameters were found to have an important effect on AHR. Pacing lead location was seen to be the strongest effect detected in our analysis in either virtual cohort, based on the number of inter-group difference tests described in Section 2.3 passed.

This result was supported by an analysis of the range of AHR possible in isolated models by changing a single model component. Changing the pacing location yielded the largest AHR change when averaged across all models in VPC2, of 18.4%, whereas the next largest, the mechanical parameters, had an average range of 12.1%.

**Fig. 7:**
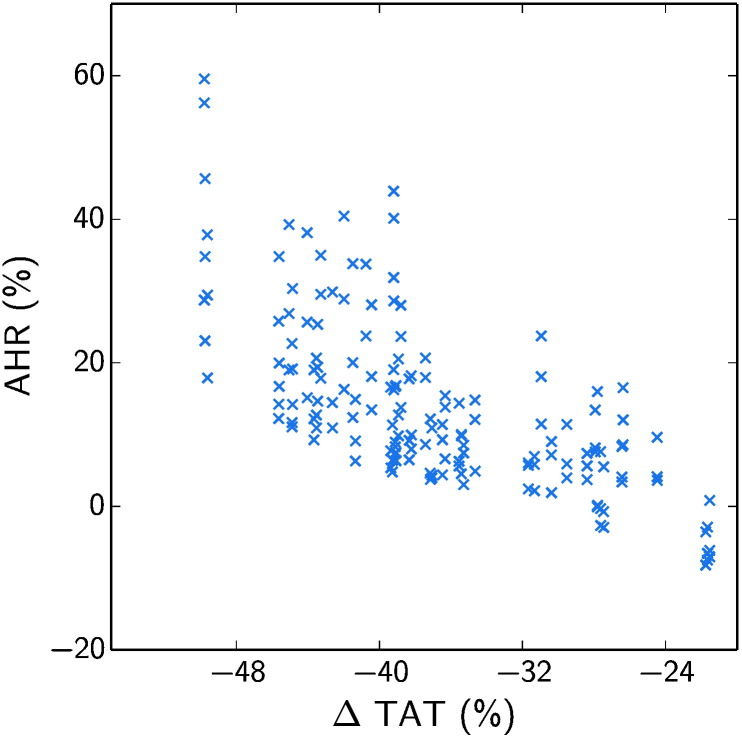
Correlation between changes in TAT on pacing and AHR for all models in the second virtual patient cohort (VPC2). A negative correlation was found, with a Spearman's rank correlation coefficient of − 0.73.

While ΔTAT was not found to be strongly affected by the presence of conduction block in VPC2, it was found that it still had a strong effect on AHR. In addition, we observed that cardiac anatomy and in particular the pacing lead locations had a strong effect on both ΔTAT and AHR. In order to consider the potential link between effects on ΔTAT and AHR, we analysed the correlation between these metrics. Fig. 7 shows how for all models in VPC2 there was a strong negative correlation between the change in TAT on pacing and AHR, with a Spearman's rank correlation coefficient of − 0.73.

## 4 Discussion

Through the creation of two virtual patient cohorts by combining components of three personalised electromechanical models of CRT, we have analysed the effect of cardiac anatomy, pacing lead location, myocardial conductivity and stiffness, afterload, active contraction and conduction block on simulated acute outcomes. In VPC1, our results showed that both the presence of conduction block and active contraction model parameters have an important effect on the response to therapy, as measured by the change in TAT on pacing and by AHR. Similarly, the cardiac anatomy and pacing lead locations were both found to be important in VPC2.

### 4.1 Consequences for patient selection

The significance of the active contraction model parameters is understandable given their role in translating myocardial activation into pump function, and indeed is consistent with previous research [16]. However, since these parameters remain challenging to reliably measure in a clinical context, it is unlikely that they would be used in clinical patient selection without the addition of a comprehensive modelling study.

The importance of conduction block for predicting CRT outcomes is of more clinical utility. Patients with conduction block experienced both greater reductions in TAT on pacing and improved AHR, a result which has also been observed in the clinic [27], [28]. While in this study the presence of conduction block was established from an invasive study, preclinical assessment of activation pattern by reconstruction from body surface ECG [29] may provide an additional tool for improved prediction of patient response and selection for therapy.

The effect of cardiac anatomy on the response to therapy is less clear, as the potential variations in cardiac anatomy are clearly not fully explored by three cases. Observed differences between models of different anatomy may be partially explained by differences in heart size, however a greater diversity of cardiac anatomies is required to identify what features affect response.

It was also found that there was a negative correlation between the change in TAT with pacing and AHR. This supports the general principle of CRT, which is that electrical resynchronisation, quantifiable as a reduction in TAT with pacing, results in improved cardiac performance and reverse remodelling, as predicted by AHR [25]. However, the suitability of AHR for the prediction of long term outcomes and the link between ΔTAT and AHR remains controversial [30], [31], [32], [33].

Nevertheless, the observed relationship between ΔTAT and AHR provides an explanation for the influence of conduction block and cardiac anatomy on AHR. Both of these properties modify the distance that must be traversed by the depolarisation wavefront in each cardiac cycle, and hence affect the measured TAT and its change with pacing. Models with greater reductions in TAT with pacing then experience a greater AHR.

### 4.2 Consequences for therapy optimisation

Pacing lead locations were also found to have an important effect on both changes in ΔTAT and AHR. While the above reported effect of conduction block has significance for the selection of appropriate patients for therapy, this finding highlights the relevance of improved lead placement. The observed differences in ΔTAT and AHR by pacing lead location demonstrate the importance of optimising CRT lead placement for achieving the best possible outcome to therapy.

The effect of pacing site on AHR was again attributed to the observed relationship between ΔTAT and AHR. With optimal choice of pacing site, TAT can be reduced, and AHR consequently improved. However, while this connection is supported by some literature [32], [34], [35], it remains controversial [33], [36]. Indeed, our analysis suggests that positive AHR is not guaranteed by a reduction in TAT. While all models in VPC1 showed a positive AHR, 7.5% of models in VPC2 did not despite having a reduction in TAT of up to 27.8%, due to the confounding effects of other parameters.

These results posed the further question of whether therapy optimisation is required on a patient-specific basis, or whether there is a common optimal pacing location across cases. The most successful pacing location, that from case 1, was positioned in the lateral wall, seen both in experiments [37] and in the clinic [38] to produce the strongest pacing response. In contrast, the pacing locations from cases 2 and 3 were on the anterior wall, seen to result in weaker responses.

We compared sets of models from VPC2 which varied only by pacing location, but whose other components remained the same, and found that while the pacing location from case 1 provided the greatest AHR in 88.5% of cases, in the remaining 11.5% the location from case 3 was superior. While this echoes the results from Fig. 6, it also shows that a single pacing location was not best in all instances. This is perhaps not surprising, given the high variability in the patient population. Heterogeneity of tissue conductivity, contractility or other factors in the myocardium may make certain areas unsuitable for pacing. Indeed, in the models where the case 3 pacing location provided the best AHR, all had conduction block.

While it was not possible to determine the variability of the location of the absolute optimal pacing location in this study, our results suggest that it will not be the same in all cases, and may be dependent on specific characteristics of individual patients. In addition, since the choice of pacing location is constrained by the coronary venous anatomy when using a transvenous delivery system [38], the need for therapy optimisation on a patient-specific basis is ever more apparent.

### 4.3 Focus for maximising response

In this paper we focus on the question of whether improved patient selection or therapy optimisation is more important for maximising outcomes to CRT. While model components relevant to both selection and optimisation are important, pacing lead locations had the strongest effect on both changes in TAT with pacing and AHR. Improved treatment planning may therefore improve overall outcomes of CRT compared to improved patient selection alone. Our results suggest that lead placement optimisation should be prioritised if we are to gain the best possible benefit from CRT.

New technologies that allow CRT with multiple LV leads [39] or freedom of pacing site by pacing on the endocardium rather than in the coronary vasculature [40] may also benefit from this conclusion. The variation of CRT efficacy with pacing site could be readily exploited by these technologies, which have greater flexibility to achieve optimal therapy outcomes in individuals and the population.

### 4.4 Limitations

The models provide a simplified representation of patient cardiac function. Fig. 2b shows that the model provided a relatively poor approximation of diastole, while providing a better description of systole, which was the period of interest in this study. The findings here are generated from three patients who had high quality and detailed experimental measurements, which limited the development of a larger number of personalised models. However, the analysis of three personalised models represents a significant advance in the coverage of the patient population when compared to previous work, which analysed a single patient case [16], [17]. While these patients are representative of DCM, these conclusions should be interpreted with caution for cohorts of ischaemic patients, since none of the cases considered presented scar.

The development of VPCs allowed analysis of a more diverse cohort, however it should be recognised that not all individuals are necessarily realistic. However, all models had a realistic EF for DCM at sinus rhythm of between 13% and 26%, offering a reasonable first order guarantee that the 648 generated models are valid. While the VPCs cannot be guaranteed to be statistically equivalent to an actual patient group, they provide a plausible representation of CRT patients. The conclusions drawn were observed consistently across the cohort and are therefore held to be representative.

### 4.5 Conclusions

The presence of conduction block was identified as an indicator for response to CRT, highlighting its potential utility for patient selection alongside other criteria. Pacing lead locations were also found to strongly affect response to therapy, which demonstrated the importance of the optimisation of CRT lead placement. While model components relevant to both patient selection and lead optimisation affected response to CRT, pacing lead locations were found to have the strongest influence. We therefore concluded that improved treatment planning should be prioritised in order to maximise CRT outcomes.

## Disclosures

SAN receives funding from Boston Scientific. CAR receives research funding and honoraria from St. Jude Medical, Medtronic and Boston Scientific.

## Figures and Tables

**Table 1:** Demographics and baseline clinical indices for the patients in this study. (QRSd: QRS duration, EF: ejection fraction, EDV: end diastolic volume)

Cases	Sex	Age	QRSd (ms)	EF (%)	EDV (ml)
1	M	63	188	23.5	310
2	F	81	139	24.7	172
3	M	77	171	19.6	331

## Appendix A Supplement: Model and Personalisation Workflow Detail

Supplement: Model and Personalisation Workflow Detail
