# Supplementary material for: The relative role of patient physiology and device optimisation in cardiac resynchronisation therapy: A computational modelling study
Source: J Mol Cell Cardiol. 2016 Jul;96:93–100. doi: 10.1016/j.yjmcc.2015.10.026 (PMC4915816; doi:10.1016/j.yjmcc.2015.10.026)
Supplement: Supplementary file 1 — Supplement: Model and Personalisation Workflow Detail [file mmc1.pdf]

The relative role of patient physiology and device optimisation in cardiac resynchronisation therapy: A computational modelling study

Online Supplement

This supplement provides a detailed account of the computational model used in the above article, and the process for its personalisation to clinical data.

1 Computational Model

We developed a weakly coupled [1] model of cardiac electromechanics, combining four main components:

- a monodomain model of electrophysiology, using the ten Tusscher cell model [2]
- a hyperelastic representation of mechanics, using the Guccione constitutive law [3]
- a phenomenological model of active contraction, based on that by Kerckhoffs *et al.* [4]
- a three element Windkessel model of afterload in ejection

1.1 Electrophysiology Model

We used a specialised electrophysiology finite element software, the Cardiac Arrhythmia Research Package (CARP) [5], for the simulation of cardiac electrophysiology. CARP is a mature and highly optimised software for bidomain and monodomain simulation of cardiac electrophysiology, developed at the Medical University of Graz (Graz, Austria) and the University of Bordeaux (Bordeaux, France).

Cardiac electrophysiology was modelled in CARP using the monodomain equation

$$\chi\left(C_m\frac{\partial V}{\partial t}+I_{\text{ion}}\right)=\nabla\cdot(\sigma\nabla V) \tag{1}$$

where χ is the cell membrane surface to volume ratio, C_m is the capacitance of the cell membrane per unit area, V is the transmembrane potential, I_{ion} is the ionic current, and σ is the tissue conductivity tensor.

The transmembrane ionic current I_{ion} was modelled with the ten Tusscher cardiac cell model [2]. This was used as although it is not the simplest available, is a sufficiently detailed human model that is computationally tractable on an organ scale when using an efficient simulation package and high performance computing (HPC) resource [6]. A tetrahedral mesh representing the anatomy of the myocardium was required, with a spatial resolution of 250 µm. This resolution represented a compromise between tractable mesh size and solution accuracy [7, 8]. Tissue conductivity was defined in the model as homogeneous across the ventricles and transversely isotropic with respect to the myofibre orientations.

1.1.1 Initial Conditions

To account for the frequency dependence of the cell model, we paced an individual cell to a limit cycle at 1 Hz for 500 beats. The isolated cell limit cycle was used as an approximation for the limit cycle in tissue. The limit cycle reached by this cell model was used as the initial state of the cell models in the whole heart simulation.

Stimuli were defined as 1 mm^3 cubes in the myocardium, representing both the intrinsic stimuli in the right ventricle (RV) and septum for left bundle branch block (LBBB) sinus rhythm and the pacing leads in cardiac resynchronisation therapy (CRT). Within the defined volumes, a stimulus current of 100 $\mu A\,cm^{-3}$ is added to the monodomain equations, initiating depolarisation of the cell model.

1.1.2 Simulation Setup and Execution

Simulations were initially run using CARP on ARCHER (http://www.archer.ac.uk/), the United Kingdom national HPC resource, using 288 cores for 2.5 − 4 hours. Activation times, as determined by the time of maximum gradient of the the transmembrane potential, were exported by the simulation software.

1.2 Mechanics Model

The model of cardiac mechanics incorporated a hyperelastic representation of passive tissue behaviour using the Guccione constitutive law [3], a phenomenological model of active ten-

sion generation based on the model by Kerckhoffs *et al.* [4] and a three element Windkessel model to represent the afterload boundary conditions.

Under this quasi-static approximation, the equations of motion to be solved are

$$\nabla \cdot \boldsymbol{\sigma} + \boldsymbol{f} = \mathbf{0} \tag{2}$$

where $\boldsymbol{\sigma}$ is the Cauchy stress tensor, and $\boldsymbol{f}$ is the body force per unit volume. It is convenient to substitute in the second Piola-Kirchhoff stress tensor $\boldsymbol{S}$, which is related to $\boldsymbol{\sigma}$ by

$$\boldsymbol{\sigma} = J^{-1}\boldsymbol{F}\boldsymbol{S}\boldsymbol{F}^T \tag{3}$$

where $\boldsymbol{F} = \frac{\partial x}{\partial X}$ is the deformation gradient tensor and $J = \det \boldsymbol{F}$ is the Jacobean determinant. In the definition of $\boldsymbol{F}$, the reference configuration $\boldsymbol{X}$ is the configuration in which the body is stress free, often referred to as the unloaded configuration, and $\boldsymbol{x}$ is the deformed, or loaded, configuration of the body. The symbols $\boldsymbol{X}$ and $\boldsymbol{x}$ here represent the state vectors of the described configurations [9]. $\boldsymbol{S}$ may then be calculated as the sum of a passive component, determined by a strain energy function $W(\boldsymbol{E})$, and an active component S_a by

$$\boldsymbol{S} = \frac{1}{2}\left(\frac{\partial W}{\partial \boldsymbol{E}} + \frac{\partial W}{\partial \boldsymbol{E}^T}\right) - P\boldsymbol{C}^{-1} + \boldsymbol{S}_a \tag{4}$$

where $\boldsymbol{C} = \boldsymbol{F}^T\boldsymbol{F}$ is the right Cauchy-Green deformation tensor, $\boldsymbol{E} = \frac{1}{2}(\boldsymbol{C} - \boldsymbol{I})$ is the Green-Lagrange strain tensor and P is the hydrostatic pressure [9].

Simulations were implemented using Continuum Mechanics, Image analysis, Signal processing and System Identification (CMISS) (http://www.cmiss.org/), developed at the University of Auckland (Auckland, New Zealand). CMISS is a mature, parallelised code, and was already capable of solving large deformation cardiac mechanics with the Guccione model.

Cardiac mechanics were solved using tricubic Hermite basis functions to describe the geometry and displacements of the myocardium. Cubic Hermite elements provide a succinct, C^1 continuous description of the cardiac anatomy. In addition, when the incompressibility constraint is added to the model, an additional hydrostatic pressure field must be introduced to the constitutive equations. The interpolation scheme must be of a lower order than that of the geometry, so a cubic basis permits use of a linear basis for the hydrostatic pressure variable, and thus continuous stresses across element boundaries [10].

1.2.1 Passive Tissue Model

To model the passive behaviour of the myocardium, we used a modified Guccione constitutive law [11], which considers muscle tissue to be hyperelastic and anisotropic with principal components aligned with the myofibre structure. The strain energy W is given by

$$W = C_1 \left(e^Q - 1 \right) \tag{5a}$$

where

$$Q = C_2 E_{\mathrm{ff}}^2 + C_3 \left(E_{\mathrm{ss}}^2 + E_{\mathrm{nn}}^2 + 2E_{\mathrm{ns}}^2 \right) + 2C_4 \left(E_{\mathrm{fs}}^2 + E_{\mathrm{nf}}^2 \right) \tag{5b}$$

E_{ff}, E_{ss} and E_{nn} are the components of the Green-Lagrange strain tensor $\boldsymbol{E}$ in the fibre, sheet and sheet normal directions, respectively, and E_{ns}, E_{fs} and E_{nf} are the corresponding shear strains. C_i ($i \in \{1, 2, 3, 4\}$) are the model parameters.

1.2.2 Active Tension

We used a phenomenological model of active tension, originally developed by Kerckhoffs *et al.* [4], and modified to reduce the number of parameters while maintaining relevance to tension generation and systolic function [12]. The active tension in the direction of the myocardial fibres T_a was given by

$$T_a = \begin{cases} T_0 \phi \tanh^2 \left(\dfrac{t_c}{t_r} \right) \tanh^2 \left(\dfrac{t_{\max} - t_c}{t_d} \right) & \text{if } 0 < t_c < t_{\max} \\ 0 & \text{otherwise.} \end{cases} \tag{6a}$$

where t_c is the time after the onset of contraction, ϕ is the nonlinear length dependent function

$$\phi = \tanh(a_6(\lambda - a_7)) \tag{6b}$$

in which λ is the stretch ratio in the fibre direction, and t_r, which regulates the rise time of the tension transient, is given by

$$t_r = t_{r0} + a_4(1 - \phi) \tag{6c}$$

There are 7 parameters in this model; the peak isometric tension T_0, the duration of tension generation $t_{\max}$, the baseline upstroke time constant t_{r0}, the downstroke time constant t_d, the length dependence of the upstroke time constant a_4, the degree of length dependence a_6, and the relative sarcomere length where no active tension is generated a_7.

The time of mechanical contraction was determined by mapping local activation times from the model of cardiac electrophysiology to the mechanics mesh, plus a fixed electromechanical delay, which is the delay from the depolarisation of the myocyte cell membrane to the start of tension generation in the myofibres. The local time relative to the onset of mechanical contraction t_c was therefore calculated by

$$t_c = t - (t_a + t_{\text{delay}}) \tag{7}$$

where t_a is the local activation time, t_{delay} is the electromechanical delay, and t is the current time in the simulation relative to the onset of ventricular depolarisation. Local activation times t_a were mapped from the nodes of the tetrahedral mesh used for electrophysiology to the Gaussian quadrature points of the cubic Hermite mesh using a nearest neighbour approach.

Active tension was included in the large deformation mechanics equations by adding it into the second Piola-Kirchhoff stress tensor as shown above in Eq. 4.

1.2.3 Boundary Conditions

Isovolumetric constraints were imposed on the ventricular cavities during the isovolumetric contraction (IVC) and isovolumetric relaxation (IVR) phases. In ejection, the three element Windkessel model was used to regulate outflow in a physiological way. This model is described by the ordinary differential equation (ODE)

$$\frac{dU}{dt} = \frac{P}{ZRC} + \frac{1}{Z}\frac{dP}{dt} - \left(\frac{1}{ZC} + \frac{1}{RC}\right)U \tag{8}$$

where U is the flow rate of blood out of the ventricle, P is the ventricular pressure, Z is the aortic resistance, and R and C are the resistance and compliance of the peripheral arterial circulation respectively. As we were primarily interested in systolic function in this study, we adopted a simplified and fast model of diastole to allow us to complete the full heart cycle. The ventricular pressure is set with a prescribed functional form.

In addition to the above ventricular cavity boundary conditions, we restrict the motion of the nodes at the base of the computational mesh. With the mesh aligned such that the base lies in the xy plane, motion of the base in the z direction is prevented by constraining both the nodal displacements and out of plane derivatives of the displacement field. This represents the effect of the stiff valve plane on cardiac mechanics and significantly improves the numerical stability of the model. Two nodes are also restricted in their motion within the plane, to prevent

arbitrary rigid body motion and rotation, without imposing artificial constraints on the solved state.

1.2.4 Solution Procedure

Simulations of cardiac mechanics with CMISS were executed on the HPC resource at the Department of Biomedical Engineering at King's College London, using 4 cores for 14 – 20 hours.

2 Personalisation Workflow

The personalisation of our model of cardiac electromechanics involved many steps, but can generally be broken down into three major sections; generation of a personalised cardiac geometry, personalisation of electrophysiology model inputs and parameters, and fitting of mechanics model inputs and parameters. A breakdown of the whole workflow can be seen visually in Fig. 1.

2.1 Geometry Personalisation

Our model of cardiac electromechanics, as introduced in Section 1, used the finite element method to solve the equations describing cardiac electrophysiology and mechanical contraction. The first part of our personalisation workflow was therefore the generation of a computational mesh which is anatomically accurate and patient specific. As described in this section, this was derived from the patient's anatomical magnetic resonance imaging (MRI), which provides a detailed description of the ventricular anatomy.

2.1.1 MRI Segmentation

The anatomical MRI was segmented, producing a binary image stack of the ventricles. Segmentation was performed either using itk-SNAP [13], an open source application for manual or semi-automatic segmentation of 3D medical images, or later in the project was performed using a fully automatic segmentation software developed by our collaborators at University College London [14]. As can be seen in Fig. 2, the automated tool segmented out many other regions of the heart, but a simple postprocessing step allowed a binary image of the myocardial volume to be generated.

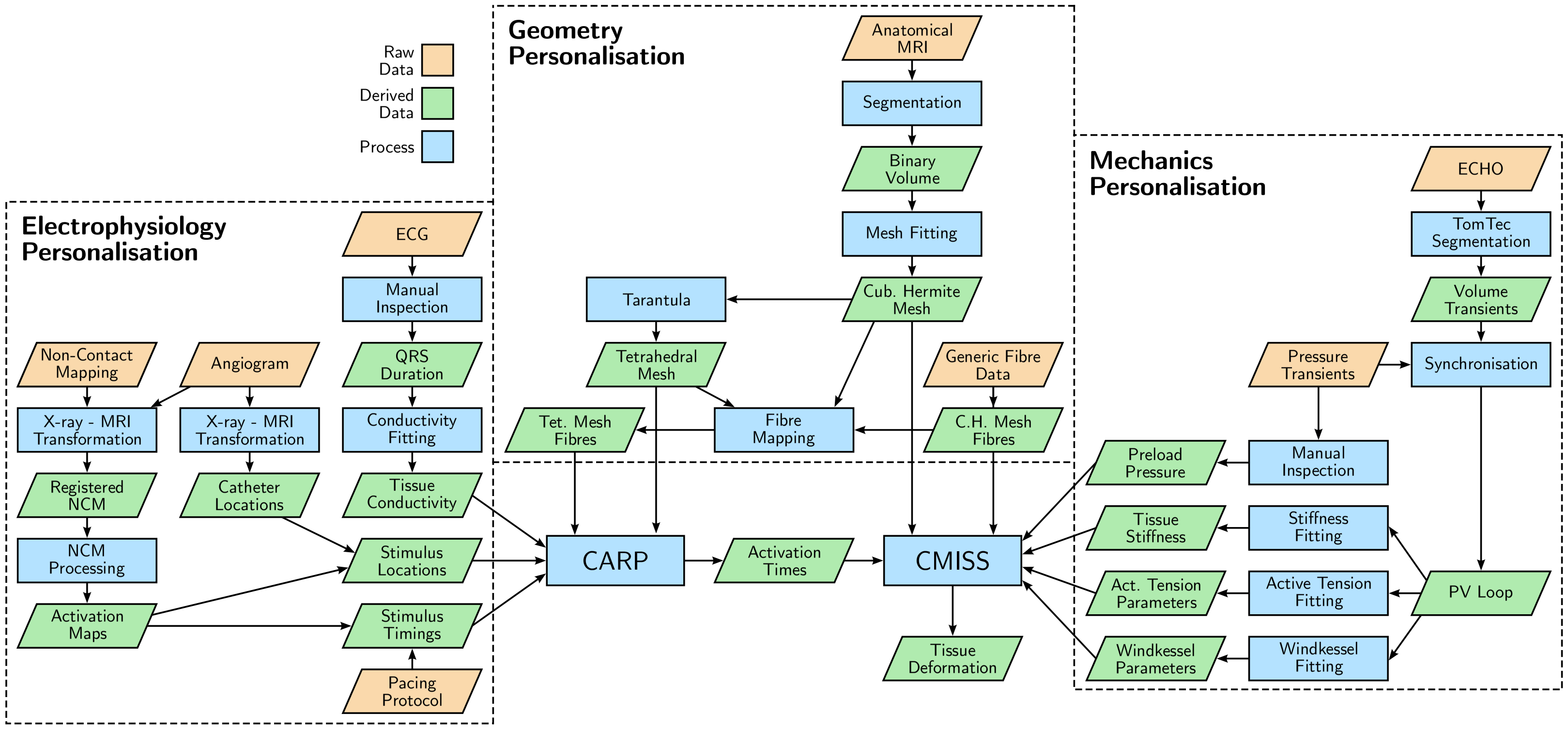

Figure 1: A flow chart of the personalisation workflow used in this project. As can be seen here, the various clinical data were processed before integration into the model. See Sections 2.1, 2.2 and 2.3 for detail of processing steps in geometry, electrophysiology and mechanics personalisation respectively.

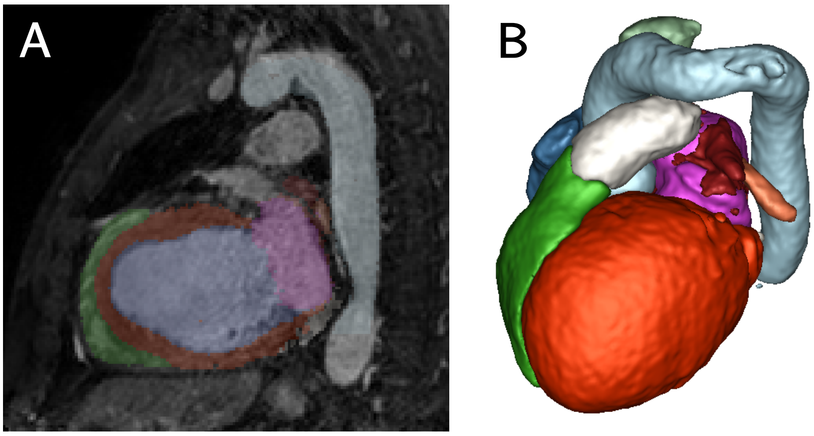

Figure 2: Automated segmentation of cardiac MRI [14]. Predefined anatomical regions were assigned different tags (a), resulting in a detailed structural model of the heart (b).

2.1.2 Mechanics Mesh Generation

The next step in the geometry personalisation process was the generation of a finite element mesh. In our workflow, we used a cubic Hermite mesh for the simulation of cardiac mechanics. Meshes were generated using the automated cubic Hermite fitting application developed by Lamata *et al.* [15, 16].

Briefly, a template mesh with a regular ellipsoidal shape was aligned with the segmentation of the myocardium. This template was then warped to match the segmentation, as illustrated in Fig. 3.

This was achieved by creating a binary image stack of the domain of the mesh, then calculating a warping field between the rasterised mesh volume and the binary segmentation using the Sheffield Image Registration Toolkit (ShIRT) [17]. This warping field was then assimilated back on to the template mesh using a variational technique described in [15]. This process was repeated several times in an iterative approach, successively improving the match between mesh and segmentation. Once this personalisation process was complete, a final post-processing step cropped the mesh to ensure a flat base plane, facilitating the later imposition of boundary conditions in simulations.

2.1.3 Electrophysiology Mesh Generation

Relatively coarse, high order meshes created by the method described in Section 2.1.2 are suitable for the simulation of large deformation mechanics, where stress and strain fields are

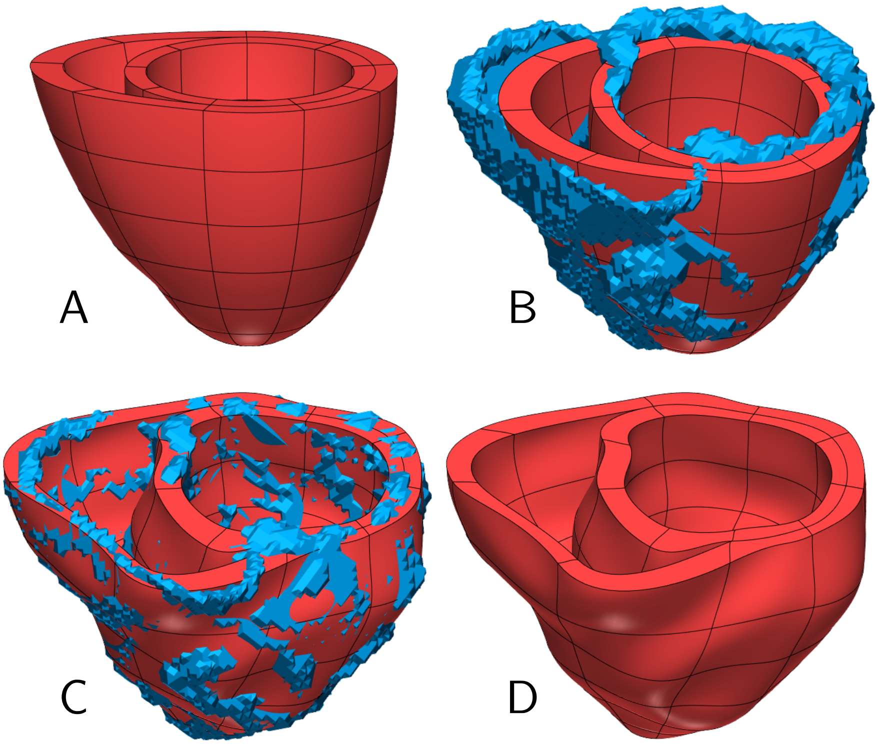

Figure 3: Customisation of a template mesh to the binary segmentation. An ellipsoidal template mesh (a) was aligned with the segmentation (b), then warped to match its geometry using the technique described in [15, 16] (c). The fitted mesh is shown in panel (d).

generally smoothly varying, but simulation of cardiac electrophysiology requires a mesh with a much finer spatial resolution. Close to the wavefront of electrical activation, there are sharp spatiotemporal gradients in the transmembrane potential which must be resolved. Previous studies have shown that sub-millimetre spatial resolution is required to achieve convergence in the simulation result when using the monodomain equations [8, 18]. For this reason, we generated a separate, high resolution mesh tailored for simulation of cardiac electrophysiology.

Meshes were generated by first rendering the fitted cubic Hermite mesh as a binary image volume with a resolution of 200 µm. The meshing package Tarantula (http://www.meshing.at/) used this binary image volume to create a high resolution (mean 250 µm edge length) tetrahedral mesh of the ventricles. As Tarantula also meshed the ventricle cavities and the region surrounding the heart, we finally postprocessed the output mesh to remove non-myocardium

elements, leaving a high quality tetrahedral mesh of the ventricles.

2.1.4 Fibre Mapping

Imaging of myofibre orientations *in vivo* was not available in our patient cohort. To approximate the heterogeneous fibre distribution that has been observed across the myocardium, we introduced rule based fibre field orientations based on human [19] and canine [20–22] measurements. Setting up fibre directions in the model was therefore not in the true sense a personalisation step, but the fibre orientation was dependent on the patient geometry and so a personalised rule based fibre field had to be generated for each patient.

In the mechanics mesh, fibre orientations were defined by angles relative to the local ξ coordinates of the finite element mesh, corrected to ensure orthonormality. The angles used are given below in Tab. 1. These angles were interpolated across the elements of the mesh with linear basis functions. Interpolating angles relative to local ξ coordinates rather than using Cartesian vectors has the advantage that their orientation can also be easily evaluated in deformed configurations.

Table 1: Generic fibre angles from human and canine data [19–22] used in the model. The fibre direction was defined at the following angles to the anticlockwise circumferential direction (ξ_1), when viewed from the basal direction. Positive angles indicate fibre directions towards the base.

Region	Angle (degrees)		
	Endo	Mid	Epi
LV free wall base	60	0	−60
LV free wall apex	83	24	−35
LV septum base	60	-	-
LV septum apex	83	-	-
RV free wall base	60	-	−60
RV free wall apex	60	-	−35
RV septum base	−60	-	-
RV septum apex	−60	-	-

On the electrophysiology mesh, fibre orientations were mapped from the mechanics mesh. To determine the fibre direction for each element, its centroid was evaluated and the corresponding element and local ξ coordinate in the mechanics mesh calculated. The fibre angles were then interpolated at that point and the corresponding Cartesian vector evaluated.

2.2 Electrophysiology Personalisation

We personalised and solved our model of cardiac electrophysiology independently of mechanics. This therefore forms the second major part of our personalisation workflow. Before the inputs and parameters of the model were personalised, the clinical data to be used for this process was processed into a more directly useful format.

2.2.1 X-Ray – MR Image Fusion

We used X-ray images from angiography to determine the positions of pacing and non-contact mapping (NCM) study catheters. The specialised X-ray – MRI (XMR) setup used in these clinical cases allowed catheter locations to be transformed into the MRI scanner coordinate space, and thus with our computational meshes.

This registration work was done previously by our colleagues in the Biomedical Engineering department at KCL [23–25]. Provided with catheter positions from the X-ray images, and the appropriate transformation matrices to MRI scanner coordinates, the pacing catheters and NCM potential maps can be visualised along with our computational mesh, as illustrated in Fig. 4.

2.2.2 Non-Contact Mapping Processing

The EnSite NCM system (St. Jude Medical, St. Paul, MN, USA) provided the functionality of exporting virtual endocardial potential traces and endocardial geometry, but not maps of depolarisation time. However, due to previously completed work on this data set [26], we were able to calculate these activation time maps from the potential traces.

The virtual potential traces were processed using one of two algorithms. The first calculated the activation time for each virtual potential trace in isolation by finding the time of the maximum positive gradient of the signal. This was known as the unipolar method. The second algorithm, known as the Laplacian method, constructed a discrete Laplacian operator using the NCM geometry and applied it to the virtual potential signals. The time of the maximum Laplacian of the signal was taken as the activation time. Fig. 5 illustrates the outcome of this processing.

Where the Laplacian algorithm produced reasonable results, with an activation pattern consistent with a visual inspection of the potential maps over time, they were used in the remainder of the workflow. Otherwise, the unipolar algorithm was used, which generally produced less

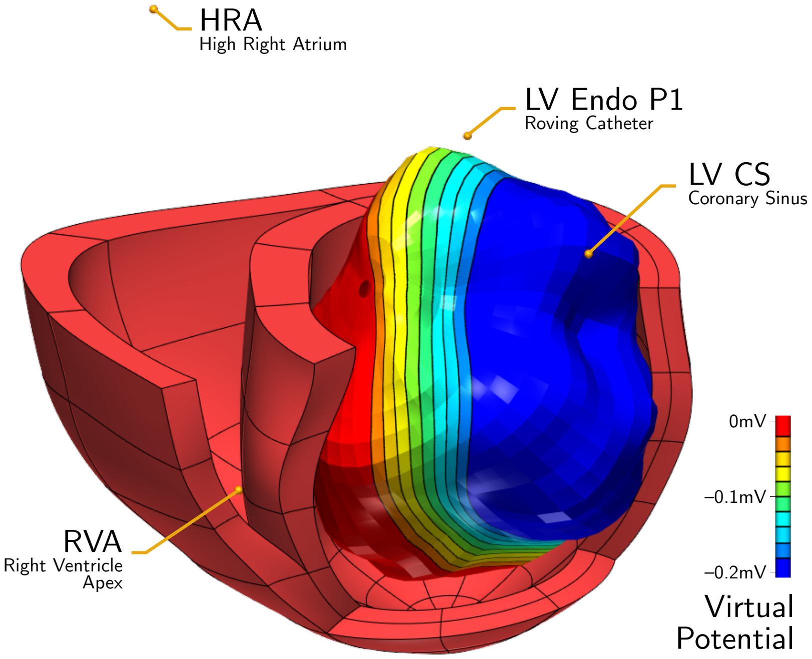

Figure 4: Registration of catheter locations and virtual endocardial surface from NCM with a personalised finite element mesh (red, anterior wall removed). Such a registration allowed the integration of real pacing sites and endocardial potential information into the model. Colours shown on the NCM geometry represent the virtual unipolar potential, tuned to highlight the propagating depolarisation wavefront from left to right.

spatially smooth results but was more robust in matching the observed activation pattern.

2.2.3 Stimuli

Electrical stimuli were added to the model for both intrinsic and paced activation sites, and were defined as small (1 mm^3) cubes embedded in the appropriate side of the ventricle wall. The location and relative timing of these stimuli were determined as detailed below. Stimulus times were determined relative to sinoatrial (SA) activation as a reference point, although as we did not model the atria only the relative timing of the stimuli was included in the simulation.

Intrinsic Activation

For intrinsic activation, we did not explicitly model the Purkinje network, but instead defined activation sites at locations in the RV free wall and septum believed to capture its effect.

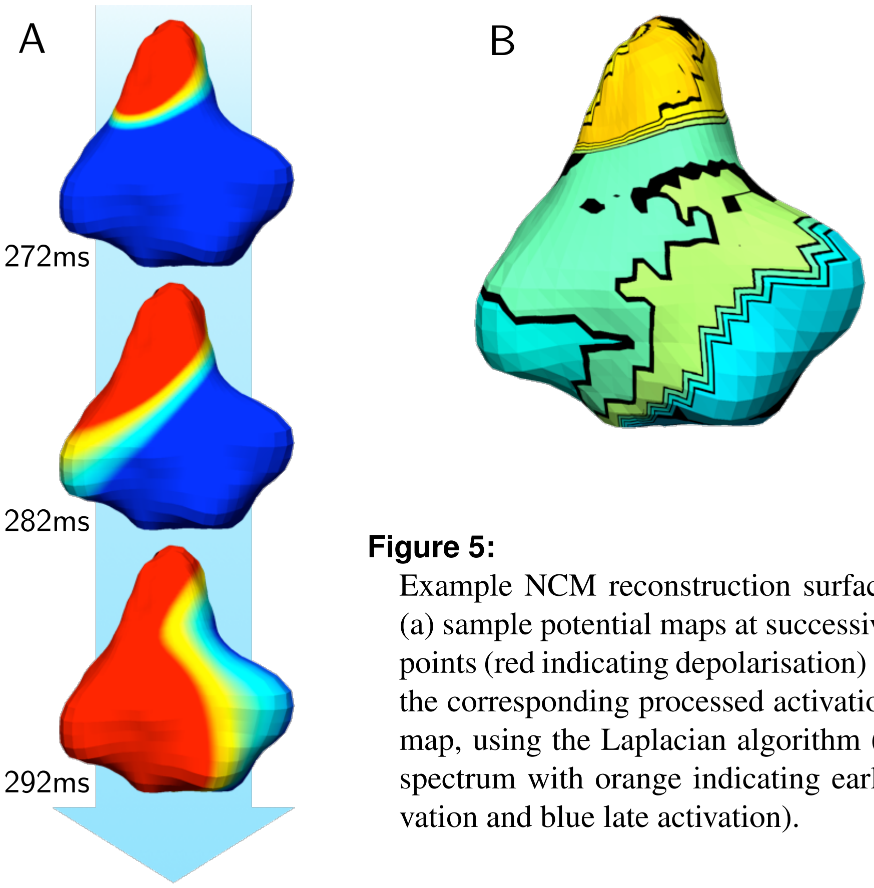

Figure 5:
Example NCM reconstruction surface with (a) sample potential maps at successive time points (red indicating depolarisation) and (b) the corresponding processed activation time map, using the Laplacian algorithm (colour spectrum with orange indicating early activation and blue late activation).

Patient specific information describing the location of the earliest site of activation in the RV was not available, so the intrinsic RV free wall activation site was estimated from an electrophysiological study of isolated human hearts in the literature [27]. In our model the time of stimulation of this point was chosen to correspond to the beginning of the QRS complex on electrocardiogram (ECG), which indicates ventricular depolarisation. In the septum, we added a stimulus at the earliest location and time of depolarisation, as seen on NCM at sinus rhythm.

Pacing Leads

The locations of pacing leads from XMR image fusion were used to choose appropriate stimulus sites in the model. During standard biventricular pacing, the coronary sinus (CS) and right ventricle apex (RVA) leads were paced 100 ms after SA node pacing by the high right atrium (HRA) lead. The relative timing of the ventricular pacing leads with the HRA lead allowed synchronisation of the paced and intrinsic stimuli.

An example of personalised intrinsic and paced stimulus locations is shown in Fig. 6.

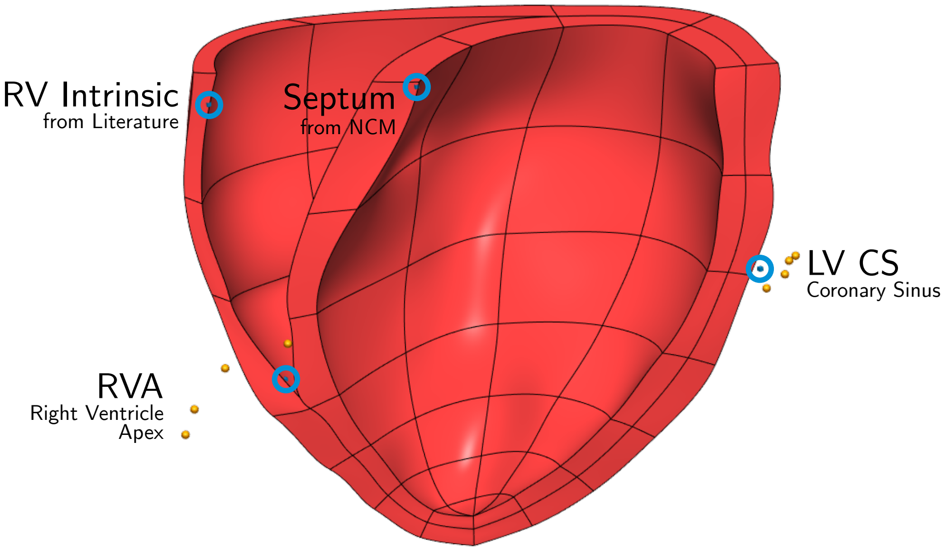

Figure 6: Stimulus volumes were defined as small (1 mm^3) cubes (blue) embedded in the myocardium (red). The septum and RV intrinsic activation sites were derived from NCM and literature respectively, while the LV and RV pacing locations were placed based on X-ray derived catheter locations (yellow).

2.2.4 Conduction Block

None of the patients in this modelling study exhibited scar on MRI, so this did not need to be incorporated into the model. However, in some cases NCM revealed a long, narrow region on the anterior wall of the LV through which the activation wave did not propagate. Instead, the activation wave had to take the slower path around this block region.

These regions appeared to be consistent between the various activation modes for which data was available. In order to characterise this behaviour in our model, we defined thin transmural regions of very low tissue conductivity. An example of such a block region is shown in Fig. 7.

2.2.5 Tissue Conductivity

In order to personalise the model of cardiac electrophysiology to each patient, we fitted the tissue conductivity to the available electrophysiological data. Activation maps did not show continuous progression of the activation wave, however noise in the signal attributable to

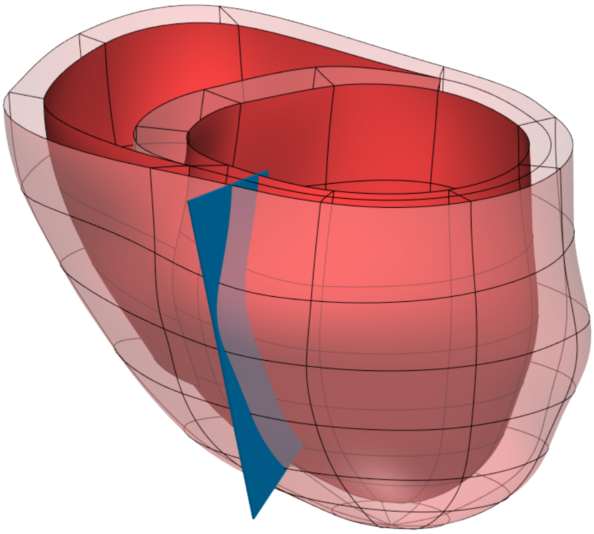

Figure 7: An example of a low conduction region to replicate electrical block observed on NCM. All elements inside the blue slab were assigned a very low conductivity.

catheter motion artefacts and dilated LV size meant that we did not have sufficient confidence in the measurements to fit a regional conduction value.

To mitigate the impact of signal noise a single conductivity value was fitted across the myocardium. We modelled the tissue conductivity as transversely isotopic with respect to the myofibre orientations. We assumed a fixed ratio of fibre to cross fibre conductivity based on previous measurements in [28], as fitting both conductivities led to an under constrained optimisation problem.

The QRS duration (QRSd) was measured manually from the ECG recorded by the NCM system, using the calliper feature in the software interface. The start of the calliper was placed at the local minimum immediately before the QRS complex, or immediately before the beginning of the principal upstroke if no minimum was identified. The end of the calliper was placed by the same criteria immediately following the QRS complex, and the time duration calculated by the NCM system was recorded. QRS durations were measured for three representative beats of each activation mode, and the average was used for fitting.

The fixed ratio conductivities were then fitted by a simple algorithm based on successive linear interpolation:

1. Two initial simulations were run with longitudinal conductivities of 0.3 and 0.5 $\mathrm{S\,m^{-1}}$, encompassing a reasonable physiological range.

2. The total activation time of the ventricles was calculated from these simulations with an automatic postprocessing step, and compared with the target value (the QRS duration).

3. A new trial value of the conductivity was estimated by interpolation between the two simulations bounding the target total activation time, or extrapolation from the closest two if the target was not bounded.

4. This trial value was then simulated and the corresponding total activation time calculated.

5. Steps 3 and 4 were repeated until the estimated trial value was equal to the previous one to three significant figures.

2.2.6 Validation

Simulated activation time maps for the LV endocardium were compared qualitatively with the activation time maps from NCM. We simulated the four pacing modalities mentioned in Section 2.2.3, using the conductivities fitted at sinus rhythm. We also checked for agreement between the simulated and measured total and total LV endocardial activation times.

2.3 Mechanics Personalisation

Cardiac mechanics, including tissue passive stiffness, Windkessel model boundary conditions and active contraction model parameters, were fitted to available clinical data. However, some of this data was not directly usable in the model fitting process so first had to be processed into a more directly usable format.

2.3.1 Ventricular Volume

Volume transients were derived from a segmentation of the preclinical 3D echocardiogram (ECHO). This was done by experienced clinicians using the TomTec analysis software (TomTec Imaging Systems, Unterschleissheim, Germany), which allowed for rapid semi-automatic tracking of the LV endocardium across the whole heart cycle. The TomTec software provided useful clinical metrics such as ejection fraction, but importantly also provided a trace of the ventricle volume over time.

2.3.2 Ventricular Pressure

Ventricular pressure was recorded during sinus rhythm and each pacing protocol, with several recordings made for each mode of activation. The PhysioMon software (Radi Medical

Systems, Uppsala, Sweden) saves this data in an easily parsable format.

Each pressure recording was first split up into separate pressure beats, using markers determined by PhysioMon and exported along with the pressure data. Any ectopic beats, characterised by early/delayed onset of systole or by abnormally high or low peak pressure, were discarded, along with the the preceding and following beats. The remaining beats were resampled and averaged for each recording, providing a smoothed, representative beat for that activation mode.

2.3.3 Pressure – Volume Synchronisation

Pressure data was first synchronised with ECG by use of data recorded in the NCM study. From a manual inspection of the 3D ECHO data from which the volume traces were calculated, an offset with the peak of the R wave on ECG was determined. Once pressure and volume were synchronised with each other through the ECG, a pressure-volume (PV) loop was plotted. An example of this is shown in Fig. 8.

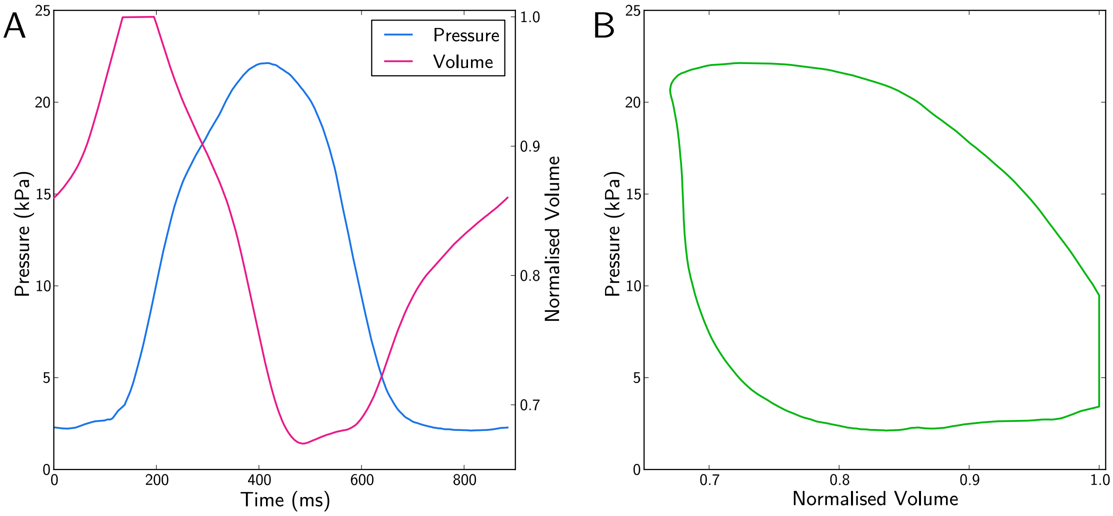

Figure 8: Result of pressure – volume data synchronisation for one patient. Once the pressure and volume traces have been synchronised in time (a), a PV loop can be plotted (b).

2.3.4 Windkessel Model

The Windkessel model was fit directly to PV data. Using the pressure transient during the ejection phase as an input, the corresponding ventricular volume was calculated by integrating

the the three-element Windkessel model ODE (Eq. 8) and the relation of outflow U to ventricular volume $U = -\frac{dV}{dt}$. These equations were solved numerically using the ODE integrator from the SciPy (http://www.scipy.org/) scientific Python library.

The three parameters of the Windkessel model were fitted by calculating a residual between the simulation and clinical volume trace. This residual was the l^2 norm of the errors between the simulation and clinical volume transients, augmented by additional constraints on the ejection fraction and duration of ejection. Importantly, a constraint forcing retrograde flow at the end of ejection was introduced. This ensured physiological behaviour in the full coupled model of electromechanics at the end of ejection, when such a flow reversal is detected and the simulation moves to the IVR phase. An example of a model fitted using the augmented constraint can be seen in Fig. 9.

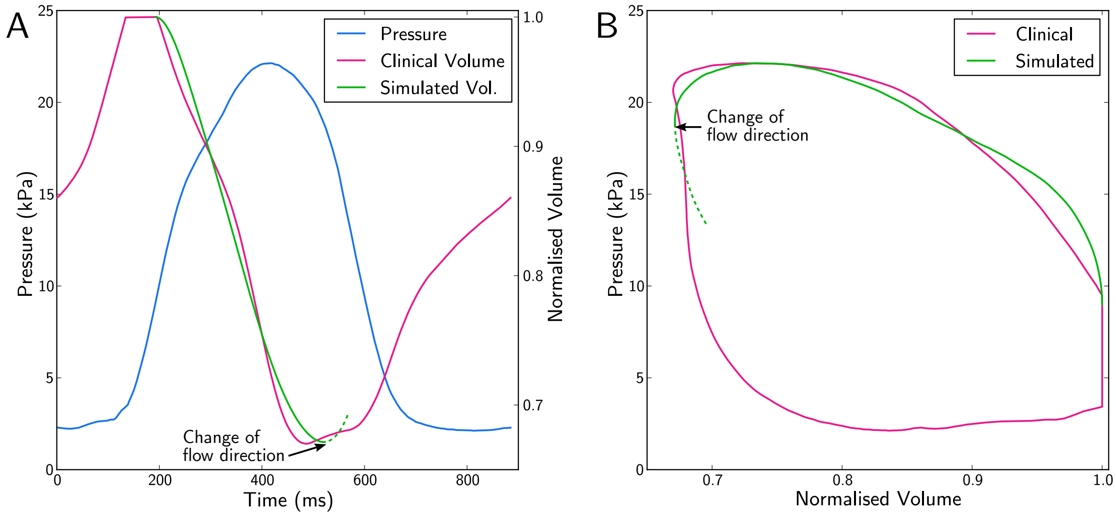

Figure 9: Example of a Windkessel model simulation with parameters fitted using the improved cost function enforcing the prediction of retrograde flow at the end of ejection. As can be seen on the simulated volume trace (a) and PV loop (b), the model predicts the flow of blood back into the ventricle from the aorta, as indicated by the dashed line. This reverse flow is not seen in a real heart cycle, as the aortic valve closes to prevent retrograde flow, and the heart enters the isovolumetric relaxation phase.

The Windkessel model parameters and the time of the start of ejection were fitted to the clinical volume trace with a combination of parameter sweeps and local refinement using the implementation of the downhill simplex algorithm in the SciPy scientific Python library. We ran a large set of optimisations from different initial guesses, which were chosen using a full

factorial experimental design.

Using this approach, Windkessel model parameters were fitted for the LV, taking around 15 minutes per case using a single core on a workstation computer. RV parameters were not personalised due to the lack of available pressure data, so were defined relative to the LV parameters using ratios sourced from canine and porcine experiments [29–31]. These ratios were 0.35 for Z, 0.125 for R and 4.5 for C.

2.3.5 Tissue Stiffness

As introduced in Section 1.2.1, the modified Guccione constitutive law by Omens *et al.* [11] has 4 parameters, C_1 to C_4, which govern the material's passive deformation. We did not have enough data to uniquely constrain the constitutive parameters, so instead assumed fixed anisotropy ratios based on previous experimental measurements in order to improve the identifiability of the parameter estimation [12]. These ratios were fixed at $C_3 = \frac{1}{2}C_2$ and $C_4 = \frac{1}{4}C_2$. This allowed us to recast the strain energy function (Eq. 5) as

$$W = C(e^{\alpha Q} - 1) \tag{9a}$$

where $C = C_1$ and

$$Q = E_{\mathrm{ff}}^2 + \frac{1}{2}\left(E_{\mathrm{ss}}^2 + E_{\mathrm{nn}}^2 + E_{\mathrm{fs}}^2 + E_{\mathrm{fn}}^2\right) + E_{\mathrm{ns}}^2 \tag{9b}$$

We then only needed to fit the two remaining parameters C and α. This was achieved by fitting the LV pressure-volume relationship to clinical data in late diastole, when the myocardium is assumed to be quiescent and we can neglect the effects of active tension.

Personalised Reference Geometry

In Section 2.1 we generated a patient specific model geometry based on the end diastolic state. However, when modelling cardiac deformations we require an unloaded, or stress free, geometry. To account for the non-zero end diastolic pressure load, we performed a deflation step to estimate the stress free configuration.

A reformulation of the finite element equations as described in [32] was used to calculate the reference state from a deformed state. In this formulation, the residual function was posed in terms of the reference state rather than the deformed state, which was then solved numerically.

Imposing the base plane boundary conditions introduced in Section 1.2.3, the reference state was calculated using the above method. Pressure data was only available for the LV so RV

pressure was approximated as 50% of the LV pressure. This was based on the ratio of diastolic pressures in the two ventricles recorded in canine experiments [33, 34] and in the clinic [35].

The solved reference configuration depended on the tissue stiffness parameters, so this step must be repeated for each stiffness we examine while fitting.

Passive Inflation

Calculation of the passive pressure-volume relationship for the ventricles was done by a passive inflation simulation. Starting from the reference state, we increased the cavity pressure in 0.2 kPa increments up to the end diastolic pressure. At each pressure level, the volume of the ventricle cavities was calculated, and the resulting pressure-volume relationship was recorded for later analysis. All reference state and subsequent passive inflation simulations were run using CMISS on a workstation computer, using 4 cores.

Parameter Fitting

To fit the constitutive law parameters in Eq. 9, we ran parameter sweeps on C and α. For each sampled pair of parameters, we calculated the associated reference configuration, and ran a passive inflation simulation up to the end diastolic state. A cost function was evaluated which sampled the PV relationship uniformly between 95% and 100% of the end diastolic volume, and computed the l^2 norm of the difference between the clinical and simulated pressures. We also included a constraint to enforce a minimum LV cavity volume in the reference configuration of $V_{50\%}$, the volume at 50% ejection ($\frac{1}{2}V_{\text{IVC}} + \frac{1}{2}V_{\text{IVR}}$).

A 5×5 full factorial parameter sweep on C and α was run, with values evenly spaced on the ranges 10 kPa to 20 kPa and 10 to 20 respectively. If a suitable match was not found on the first sweep, extensions or refinements of the sweep were run.

2.3.6 Active Tension

Active tension model parameters were fitted to the clinical pressure and volume data. Using the setup described in Section 1.2.4, simulations of cardiac electromechanics were run for given sets of active tension parameter values. The simulations each generated a file with ventricle pressures and volumes over time, facilitating a comparison with clinical data such as that seen in Fig. 10.

Note that the difference in clinical and simulated volumes in the first 150 ms in Fig. 10b is to be expected, as during this period the LV is still in diastole. Since we simulated only a

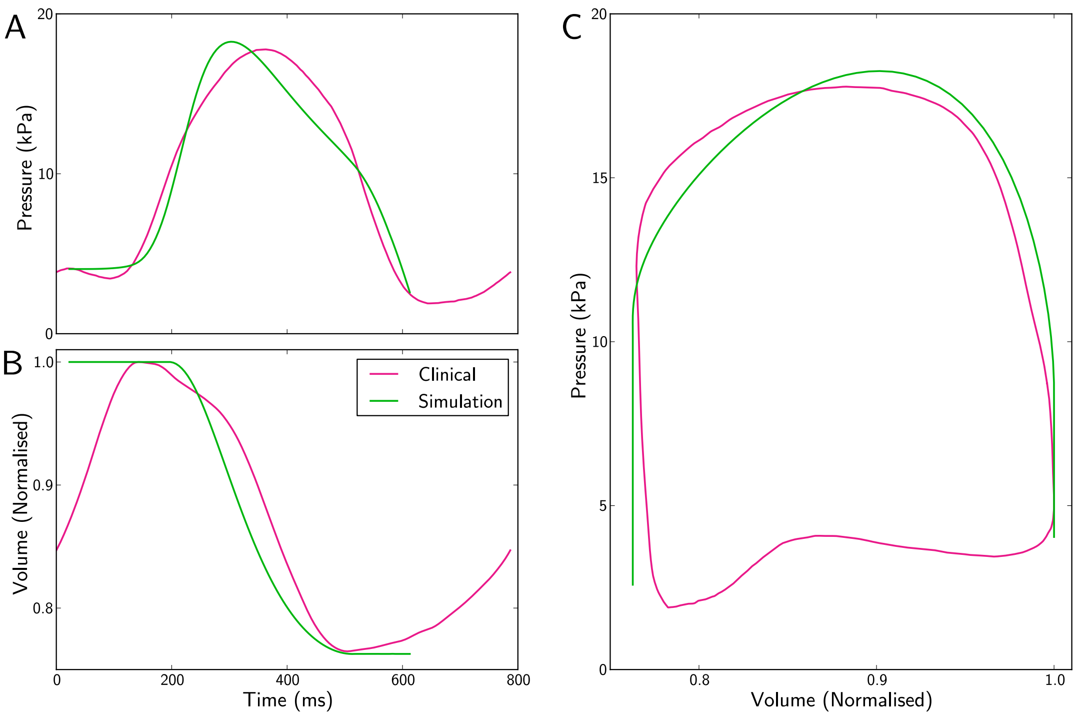

Figure 10: Comparison of clinical and simulated (a) pressure transients, (b) volume transients and (c) corresponding PV loops.

single beat, the LV was placed in its end diastolic state at $t = 0$, and remained quiescent for some time due to the dyssynchronous contraction of LBBB. The simulation was terminated at the end of IVR as diastolic passive filling does not affect the calculated cost function for active tension model fitting.

Cost Function Design

A cost function was developed based on the calculation of metrics describing geometrical features of the pressure and volume transients. The cost function targeted global features of the pressure and volume transients as a naïve l^2 norm of the difference between simulated and clinical transients was found to be overly sensitive to specific features, for example the early upstroke of the pressure transient. The calculated metrics, as seen in Fig. 11, were defined in a sufficiently robust way so that the same algorithm could be applied to both clinical and simulation data.

Our 'geometric' cost function combined these metrics $\{p_i\}$ together using the fractional difference of the simulation value with the clinical value to mitigate the effect of very different

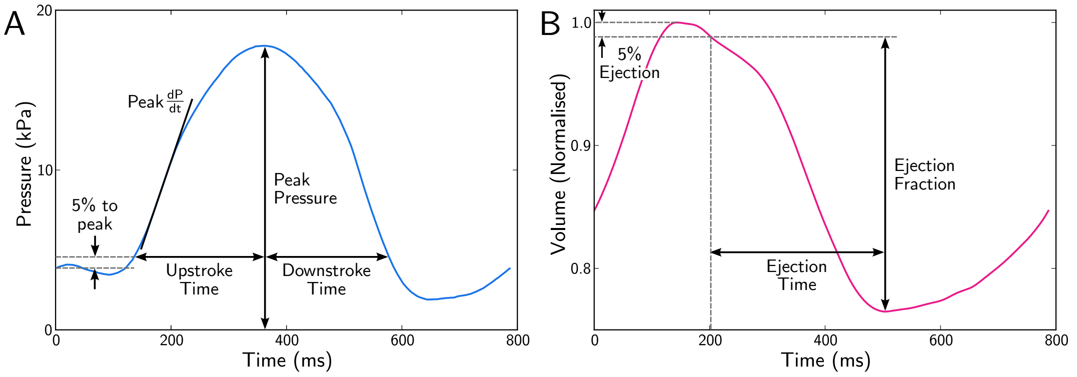

Figure 11: Calculated metrics used in the 'geometric' cost function. From the pressure transient (a), peak pressure and peak $\frac{dP}{dt}$ on the upstroke were calculated, in addition to upstroke and downstroke times, starting and finishing from the time at which pressure was 5% between the starting pressure and peak pressure. On the volume transient (b), ejection fraction and time were calculated, similarly starting from the time of 5% ejection.

magnitudes. It was computed as

$$R_g = ||\boldsymbol{r}|| \tag{10a}$$

where

$$r_i = \omega_i \frac{p_i^{\text{clinical}} - p_i^{\text{sim}}}{p_i^{\text{clinical}}} \tag{10b}$$

and $\{\omega_i\}$ are a set of weights that were manually adjusted to prioritise features deemed more important. The weights used are given in Tab. 2.

Table 2: Component weights $\{\omega_i\}$ of the 'geometric' cost function (Eq. 10). Residual components are those shown in Fig. 11.

Component	Weight ω
Peak Pressure	5.0
Upstroke Time	1.0
Downstroke Time	1.0
Peak $\frac{dP}{dt}$	1.0
Ejection Fraction	5.0
Ejection Time	5.0

It was found necessary to add an additional constraint to the cost function, including pressure data from paced activation modes. Models fitted without this data exhibited a good fit

at sinus rhythm, but failed to show any significant change on pacing. To bring our models to parameter values replicating the response of the heart to pacing, simulations of contraction under standard biventricular (BiV SIM) pacing were run and the acute haemodynamic response (AHR) calculated. AHR is computed as

$$\mathrm{AHR} = \frac{\max \frac{dP}{dt}\Big|_{\mathrm{paced}} - \max \frac{dP}{dt}\Big|_{\mathrm{baseline}}}{\max \frac{dP}{dt}\Big|_{\mathrm{baseline}}} \tag{11}$$

where $\max \frac{dP}{dt}$ is the peak rate of change of pressure on the upstroke, as seen in Fig. 11. We posed a response cost which was again the fractional difference between simulation and clinical data

$$R_r = \frac{\mathrm{AHR}_{\mathrm{clinical}} - \mathrm{AHR}_{\mathrm{sim}}}{\mathrm{AHR}_{\mathrm{clinical}}} \tag{12}$$

and combined it with the geometric cost R_g to reach the full cost function

$$R = \alpha R_g + (1 - \alpha) R_r \tag{13}$$

with the new parameter α providing control over the relative weights of the geometric and response costs.

Parameter Sweeps

Of the 7 parameters in the active tension model (see Section 1.2.2 for details), 6 were fitted to clinical data (specifically T_0, t_{r0}, t_d, $t_{\max}$, a_4 and a_6), while a_7 was fixed to an experimentally validated value of 0.7 [36].

We sampled the parameter space using Latin hypercube sampling (LHS) [37]. LHS designs have an advantage over more conventional full factorial designs in that they provide a good coverage of all parameters' ranges, and also have an advantage over simple random sampling as they ensure a more homogeneous sampling density. LHS also offers the practical advantage that the number of samples is independent of the dimensionality of the parameter space.

Fitting of the active tension model was done using an iterative approach. Initial sweeps with 150 samples were run using the parameter ranges given in Tab. 3, with later narrower sweeps refining the search.

Table 3: Initial active tension parameter sweep ranges using the LHS design.

Parameter	Range	Units
T_0	80 – 200	kPa
t_r0	10 – 100	ms
t_d	80 – 150	ms
$t_{\max}$	450 – 600	ms
a_4	200 – 1000	ms
a_6	3 – 7	1

2.3.7 Validation

Once the fitting processes outlined in this section were completed, validation was performed by checking for agreement between the simulated deformation of the heart at sinus rhythm and the pre-implantation cine MRI.

Short axis cine MRI stacks were registered with the model geometry using the embedded scanner orientation information. An image slice half way between the apex and base was selected for comparison with simulation results, and a tissue boundary contour was generated from the model in the same plane and overlayed on the image. Frames were then generated at 100 ms intervals, and a visual comparison done to validate that the model accurately reproduced ventricular deformations at sinus rhythm.

2.4 Ensuring Uniqueness of Fit

As our computational model of cardiac electromechanics has a large number of parameters and inputs, we must be careful to ensure a unique fit to model data when performing the model personalisation. The necessary complexity of the model results in a large and nonlinear parameter space within which it is not feasible to guarantee uniqueness. Our personalisation approach therefore exploits the ability to separate several parts of the model and fit them to different data or phases of the heart cycle. As explained in this supplement, tissue conductivity is fitted using ECG data (Section 2.2.5), the Windkessel model is fitted using pressure and volume transients during ejection (Section 2.3.4), passive tissue stiffness is fitted using the late diastolic pressure-volume relation (Section 2.3.5), and the model of active contraction is fitted to systolic pressure and volume transients (Section 2.3.6). This targeted use of data with the components for which they have greatest relevance increased our capacity to achieve a constrained parameter set.

Furthermore, our approach focuses on fitting those parameters that are pertinent to our application. Where data was insufficient to personalise all model parameters, values were determined using literature based measurements or ratios to other parameters in order to ensure a unique fit. This was the case for the anisotropy ratio of the tissue conductivity σ_x/σ_f (Section 2.2.5) and the sarcomere length ratio at which no active tension is generated a_7 (Section 2.3.6).

Acronyms

AHR acute haemodynamic response

CARP Cardiac Arrhythmia Research Package

CMISS Continuum Mechanics, Image analysis, Signal processing and System Identification

CRT cardiac resynchronisation therapy

CS coronary sinus

ECG electrocardiogram

ECHO echocardiogram

HPC high performance computing

HRA high right atrium

IVC isovolumetric contraction

IVR isovolumetric relaxation

LBBB left bundle branch block

LHS Latin hypercube sampling

LV left ventricle

MRI magnetic resonance imaging

NCM non-contact mapping

ODE ordinary differential equation

QRSd QRS duration

RV right ventricle

RVA right ventricle apex

SA sinoatrial

ShIRT Sheffield Image Registration Toolkit

References

[1] Steven A Niederer and Nicolas P Smith. An improved numerical method for strong coupling of excitation and contraction models in the heart. *Progress in Biophysics and Molecular Biology*, 96(1-3):90–111, 2008.

[2] K H W J ten Tusscher and A V Panfilov. Alternans and spiral breakup in a human ventricular tissue model. *American Journal of Physiology: Heart and Circulatory Physiology*, 291(3):H1088–100, 2006.

[3] J M Guccione, A D McCulloch, and L K Waldman. Passive Material Properties of Intact Ventricular Myocardium Determined From a Cylindrical Model. *Journal of Biomechanical Engineering*, 113(1):42, February 1991.

[4] R C P Kerckhoffs, P H M Bovendeerd, F W Prinzen, K Smits, and T Arts. Intra- and interventricular asynchrony of electromechanics in the ventricularly paced heart. *Journal of Engineering Mathematics*, 47(3-4):201–216, 2003.

[5] Edward J Vigmond, Matt Hughes, G Plank, and L Joshua Leon. Computational Tools for Modeling Electrical Activity in Cardiac Tissue. *Journal of Electrocardiology*, 36(Suppl.):69–74, January 2003.

[6] Steven A Niederer, Lawrence Mitchell, Nicolas Smith, and Gernot Plank. Simulating human cardiac electrophysiology on clinical time-scales. *Frontiers in Physiology*, 2:14, January 2011.

[7] R H Clayton and A V Panfilov. A guide to modelling cardiac electrical activity in anatomically detailed ventricles. *Progress in Biophysics and Molecular Biology*, 96(1-3):19–43, January 2008.

[8] Steven A Niederer, Eric Kerfoot, Alan P Benson, Miguel O Bernabeu, Olivier Bernus, Chris Bradley, Elizabeth M Cherry, Richard Clayton, Flavio H Fenton, Alan Garny, Elvio Heidenreich, Sander Land, Mary Maleckar, Pras Pathmanathan, Gernot Plank, José F Rodríguez, Ishani Roy, Frank B Sachse, Gunnar Seemann, Ola Skavhaug, and Nic P Smith. Verification of cardiac tissue electrophysiology simulators using an N-version benchmark. *Philosophical Transactions of the Royal Society A*, 369(1954):4331–4351, November 2011.

[9] Javier Bonet and Richard D Wood. *Nonlinear Continuum Mechanics for Finite Element Analysis*. Cambridge University Press, 1997.

[10] Martyn Nash. *Mechanics and Material Properties of the Heart using an Anatomically Accurate Mathematical Model*. PhD Thesis, University of Auckland, 1998.

[11] Jeff H Omens, Deidre A MacKenna, and Andrew D McCulloch. Measurement of Strain and Analysis of Stress in Resting Rat Left Ventricular Myocardium. *Journal of Biomechanics*, 26(6):665–676, 1993.

[12] Steven A Niederer, Gernot Plank, Phani Chinchapatnam, Matthew Ginks, Pablo Lamata, Kawal S Rhode, Christopher Aldo Rinaldi, Reza Razavi, and Nicolas P Smith. Length-dependent tension in the failing heart and the efficacy of cardiac resynchronization therapy. *Cardiovascular Research*, 89(2):336–43, February 2011.

[13] Paul A Yushkevich, Joseph Piven, Heather Cody Hazlett, Rachel Gimpel Smith, Sean Ho, James C Gee, and Guido Gerig. User-guided 3D active contour segmentation of anatomical structures: Significantly improved efficiency and reliability. *NeuroImage*, 31(3):1116–28, July 2006.

[14] Maria A Zuluaga, M Jorge Cardoso, Marc Modat, and Sebastien Ourselin. Multi-atlas Propagation Whole Heart Segmentation from MRI and CTA Using a Local Normalised Correlation Coefficient Criterion. In *FIMH 2013*, pages 174–181.

[15] Pablo Lamata, Steven Niederer, David Nordsletten, David C Barber, Ishani Roy, D Rod Hose, and Nic Smith. An accurate, fast and robust method to generate patient-specific cubic Hermite meshes. *Medical Image Analysis*, 15(6):801–13, December 2011.

[16] Pablo Lamata, Matthew Sinclair, Eric Kerfoot, Angela Lee, Andrew Crozier, Bojan Blazevic, Sander Land, Adam J Lewandowski, David Barber, Steve Niederer, and Nic Smith. An automatic service for the personalization of ventricular cardiac meshes. *Journal of the Royal Society Interface*, 11(91):20131023, February 2014.

[17] D C Barber, E Oubel, A F Frangi, and D R Hose. Efficient computational fluid dynamics mesh generation by image registration. *Medical Image Analysis*, 11(6):648–62, December 2007.

[18] R H Clayton, O Bernus, E M Cherry, H Dierckx, F H Fenton, L Mirabella, A V Panfilov, Frank B Sachse, G Seemann, and H Zhang. Models of cardiac tissue electrophysiology: Progress, challenges and open questions. *Progress in Biophysics and Molecular Biology*, 104(1-3):22–48, 2011.

[19] R A Greenbaum, S Y Ho, D G Gibson, A E Becker, and R H Anderson. Left ventricular fibre architecture in man. *British Heart Journal*, 45(3):248–63, March 1981.

[20] I J LeGrice, P J Hunter, and B H Smaill. Laminar structure of the heart: a mathematical model. *The American Journal of Physiology*, 272(5 Pt 2):H2466–76, May 1997.

[21] T P Usyk, R Mazhari, and A D McCulloch. Effect of Laminar Orthotropic Myofiber Architecture on Regional Stress and Strain in the Canine Left Ventricle. *Journal of Elasticity*, 61(1-3):143–164, July 2000.

[22] Yasuo Takayama, Kevin D Costa, and James W Covell. Contribution of laminar myofiber architecture to load-dependent changes in mechanics of LV myocardium. *American Journal of Physiology: Heart and Circulatory Physiology*, 282(4):H1510–20, April 2002.

[23] Kawal S Rhode, Maxime Sermesant, David Brogan, Sanjeet Hegde, John Hipwell, Pier Lambiase, Eric Rosenthal, Clifford Bucknall, Shakeel a Qureshi, Jaswinder S Gill, Reza Razavi, and Derek L G Hill. A System for Real-Time XMR Guided Cardiovascular Intervention. *IEEE Transactions on Medical Imaging*, 24(11):1428–40, November 2005.

[24] Matthew R Ginks. *Addressing Non-response to Cardiac Resynchronization Therapy: Underlying Mechanisms and New Therapeutic Approaches*. MD Thesis, King's College London, 2011.

[25] Michael V N Truong. *2D-3D Registration of Cardiac Images*. PhD Thesis, King's College London, 2014.

[26] Phani Chinchapatnam, Kawal S Rhode, Matthew Ginks, Christopher Aldo Rinaldi, Pier Lambiase, Reza Razavi, Simon Arridge, and Maxime Sermesant. Model-Based Imaging of Cardiac Apparent Conductivity and Local Conduction Velocity for Diagnosis and Planning of Therapy. *IEEE Transactions on Medical Imaging*, 27(11):1631–1642, 2008.

[27] D Durrer, R T van Dam, G E Freud, M J Janse, F L Meijler, and R C Arzbaecher. Total Excitation of the Isolated Human Heart. *Circulation*, 41(6):899–912, June 1970.

[28] Bryan J Caldwell, Mark L Trew, Gregory B Sands, Darren A Hooks, Ian J LeGrice, and Bruce H Smaill. Three Distinct Directions of Intramural Activation Reveal Nonuniform Side-to-Side Electrical Coupling of Ventricular Myocytes. *Circulation: Arrhythmia and Electrophysiology*, 2(4):433–440, 2009.

[29] W P Santamore and D Burkhoff. Hemodynamic consequences of ventricular interaction as assessed by model analysis. *The American Journal of Physiology*, 260(1 Pt 2):H146–57, January 1991.

[30] P M Heerdt, C D Gandhi, and M L Dickstein. Disparity of lsoflurane Effects on Left and Right Ventricular Afterload and Hydraulic Power Generation in Swine. *Anesthesia and Analgesia*, 87(3):511–21, September 1998.

[31] Mustafa Karamanoglu and Tom D Bennett. A Right Ventricular Pressure Waveform Based Pulse Contour Cardiac Output Algorithm in Canines. *Cardiovascular Engineering*, 6(3):83–92, September 2006.

[32] Vijayaraghavan Rajagopal, Jae-Hoon Chung, David Bullivant, Poul M. F. Nielsen, and Martyn P. Nash. Determining the finite elasticity reference state from a loaded configuration. *International Journal for Numerical Methods in Engineering*, 72(12):1434–1451, December 2007.

[33] W C Little, F R Badke, and R A O'Rourke. Effect of Right Ventricular Pressure on the End- Diastolic Left Ventricular Pressure-Volume Relationship before and after Chronic Right Ventricular Pressure Overload in Dogs without Pericardia. *Circulation Research*, 54(6):719–30, June 1984.

[34] Charles E Bemis, Juan R Serur, David Borkenhagen, Edmund H Sonnenblick, and Charles W Urschel. Influence of Right Ventricular Filling Pressure on Left Ventricular Pressure and Dimension. *Circulation Research*, 34(4):498–504, April 1974.

[35] M Mathison, J R Edgerton, J L Horswell, J J Akin, and M J Mack. Analysis of Hemodynamic Changes During Beating Heart Surgical Procedures. *The Annals of Thoracic Surgery*, 70(4):1355–60; discussion 1360–1, October 2000.

[36] J C Kentish, H E ter Keurs, L Ricciardi, J J Bucx, and M I Noble. Comparison between the Sarcomere Length-Force Relations of Intact and Skinned Trabeculae from Rat Right Ventricle. *Circulation Research*, 58(6):755–68, June 1986.

[37] M D McKay, R J Beckman, and W J Conover. A Comparison of Three Methods for Selecting Values of Input Variables in the Analysis of Output from a Computer Code. *Technometrics*, 21(2):239–245, February 2000.
